# PS-39 - ENGLAND’S LOOKBACK EXERCISE AND INCIDENT RESPONSE TO FALSIFIED RABIES VACCINE IN CIRCULATION IN INDIA

**DOI:** 10.1093/pubmed/fdag046.075

**Published:** 2026-07-09

**Authors:** Dr Shivan Thakrar, Miss Sameera Lyons, Mr Thomas Munday, Dr Katherine Russell, Dr Hannah Emmett

**Affiliations:** UK Health Security Agency; UK Health Security Agency; UK Health Security Agency; UK Health Security Agency; UK Health Security Agency

## Abstract

**Background:**

In September 2025, the UK Health Security Agency (UKHSA) was alerted to a report of a falsified batch of Abhayrab rabies vaccine circulating in India. Falsified product details included a batch number and a printed manufacture date of November 2023. Limited additional information was available. Rabies is invariably fatal once symptoms develop. While rare, cases of rabies can develop years after an exposure.

**Objectives:**

We conducted a lookback exercise to identify individuals who may have received falsified rabies vaccine and provide replacement vaccine.

**Methods:**

UKHSA formed a multi-agency incident management team that involved expertise in immunisation, travel health, and representation from Scotland, Wales, and Northern Ireland. We agreed a case definition to identify individuals at risk, who received Abhayrab rabies vaccine or an unknown brand in India alongside less than three vaccines in the UK/other locations without concerns about falsified vaccine. Cases were identified through UKHSAs national database for rabies exposures. Bespoke emails outlining UKHSA's suggested number of vaccine doses and a request for a GP-led review with patients were sent to general practices for exposures since 1st November 2023.

**Results:**

The lookback identified 327 patients. 284/327 (87%) had a complete follow up, of whom 146/284 (51%) were provided with additional vaccine. The median time to receive treatment was 21 days (range:3-101). 120/284 (43%) did not require action. 79/120 (66%) had sufficient prior vaccination history, 35/120 (29%) did not have an actual exposure and 6/120 (5%) were referred elsewhere for management. 18/327 (5%) refused vaccine. In the UK, no known rabies cases have been associated with the concerning vaccine.

**Conclusions:**

Prompt action to mitigate the risk of inadequate protection associated with falsified rabies vaccines is an important health protection function. A national database of rabies exposures in England facilitated rapid risk assessment, case identification and patient notification via primary care.

